# Supplementary figures and images for: Molecular characterization of breast and lung tumors by integration of multiple data types with functional sparse-factor analysis
Source: PLoS Comput Biol. 2018 Oct 31;14(10):e1006520. doi: 10.1371/journal.pcbi.1006520 (PMC6231682; doi:10.1371/journal.pcbi.1006520)

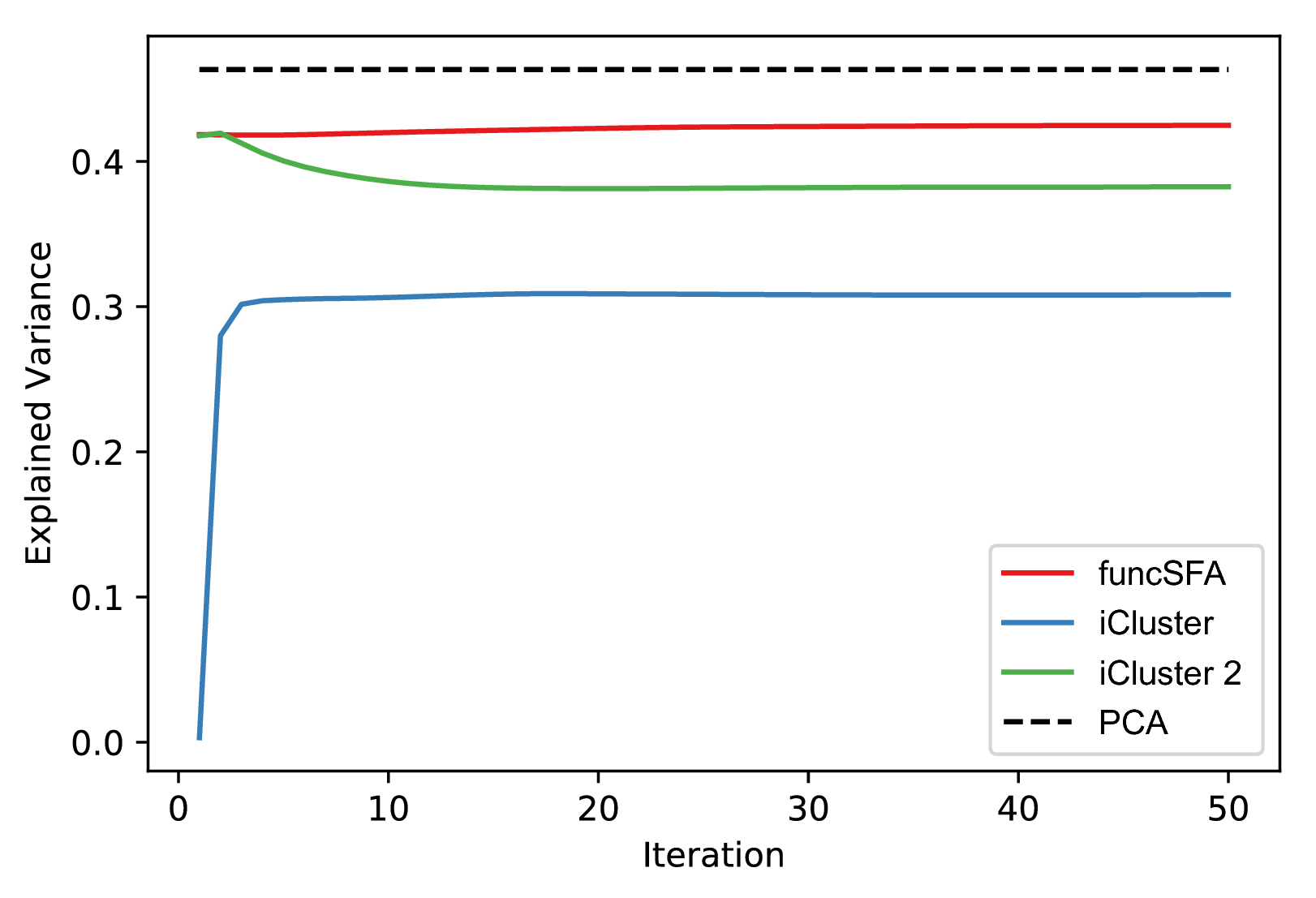

Supplement: S1 Fig — Showing the explained variance of a model over the first 50 iterations for funcSFA, iCluster and iCluster2. Best possible explained variance as determined by principal component analysis (PCA) is shown as a benchmark. (TIF) [file pcbi.1006520.s006.tif]

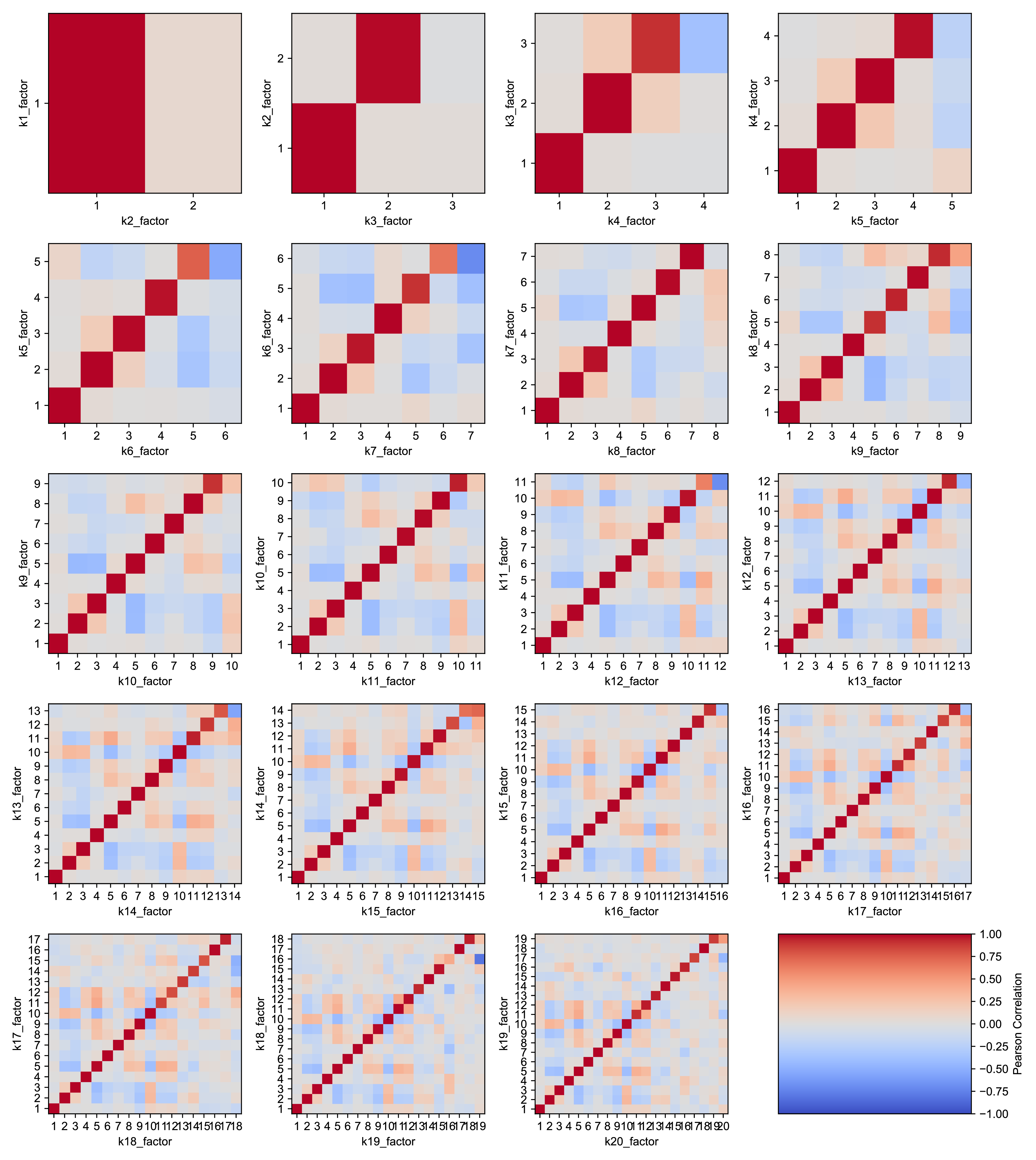

Supplement: S2 Fig — (TIF) [file pcbi.1006520.s007.tif]

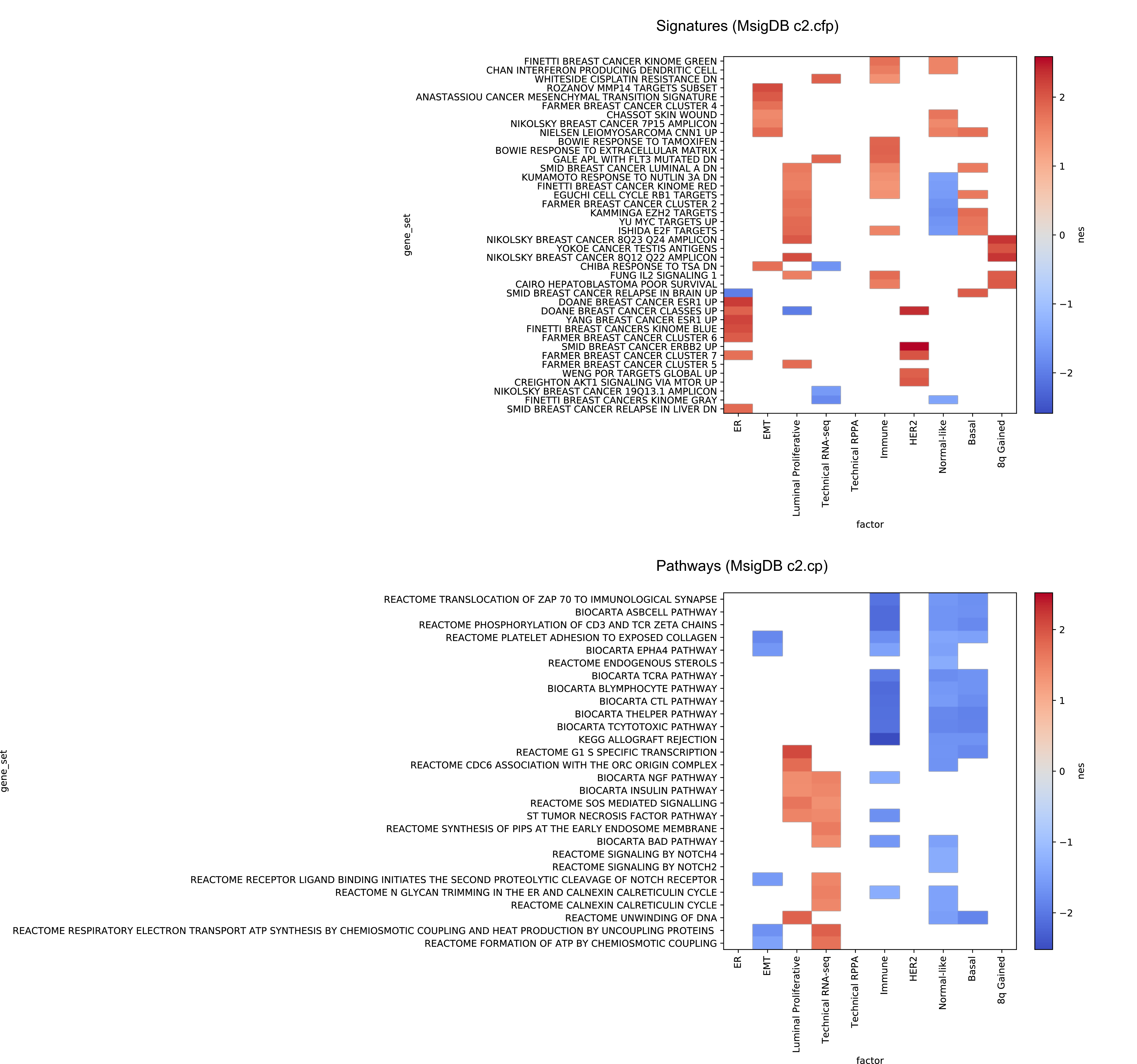

Supplement: S4 Fig — (TIF) [file pcbi.1006520.s009.tif]

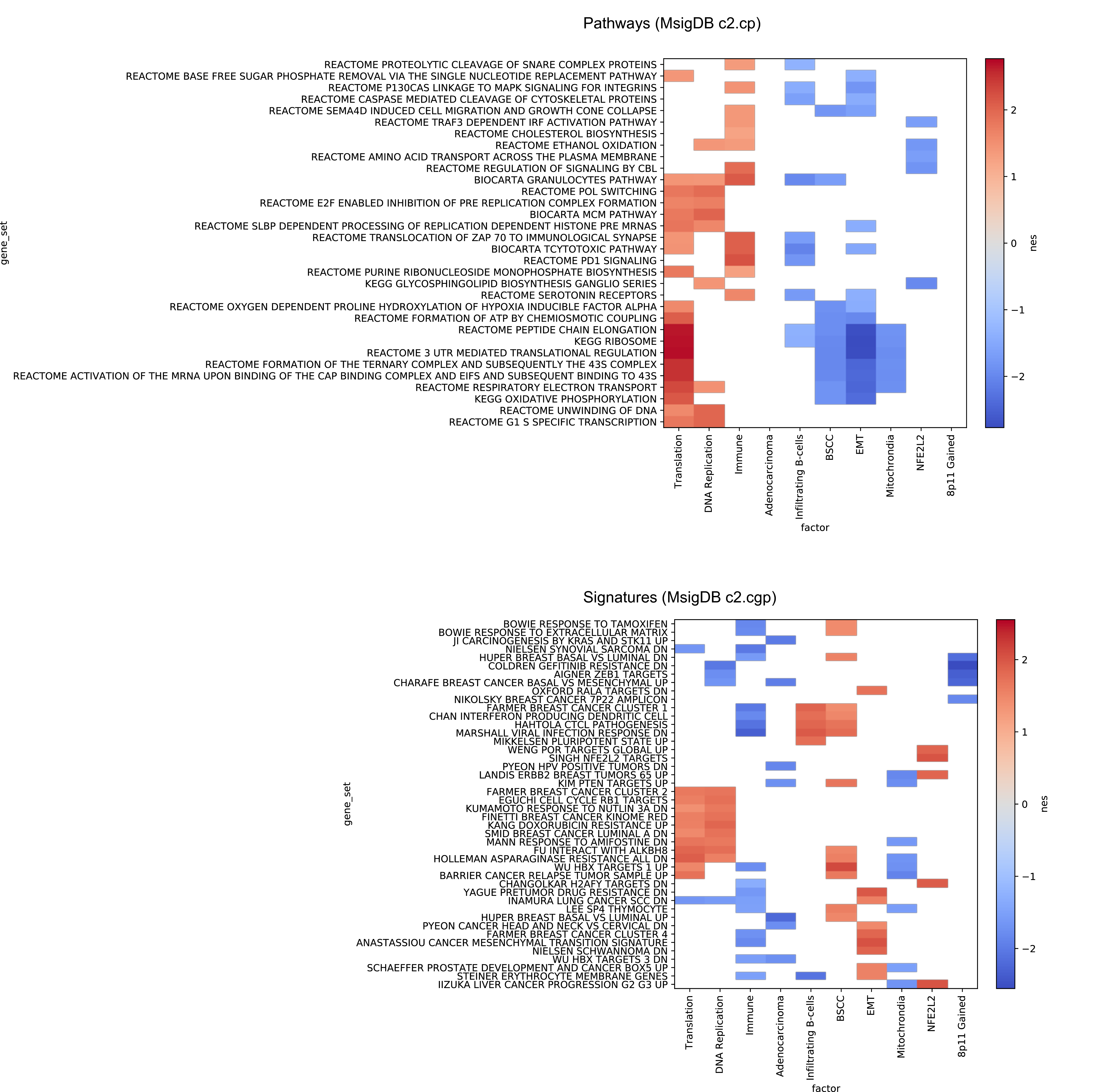

Supplement: S5 Fig — (TIF) [file pcbi.1006520.s010.tif]

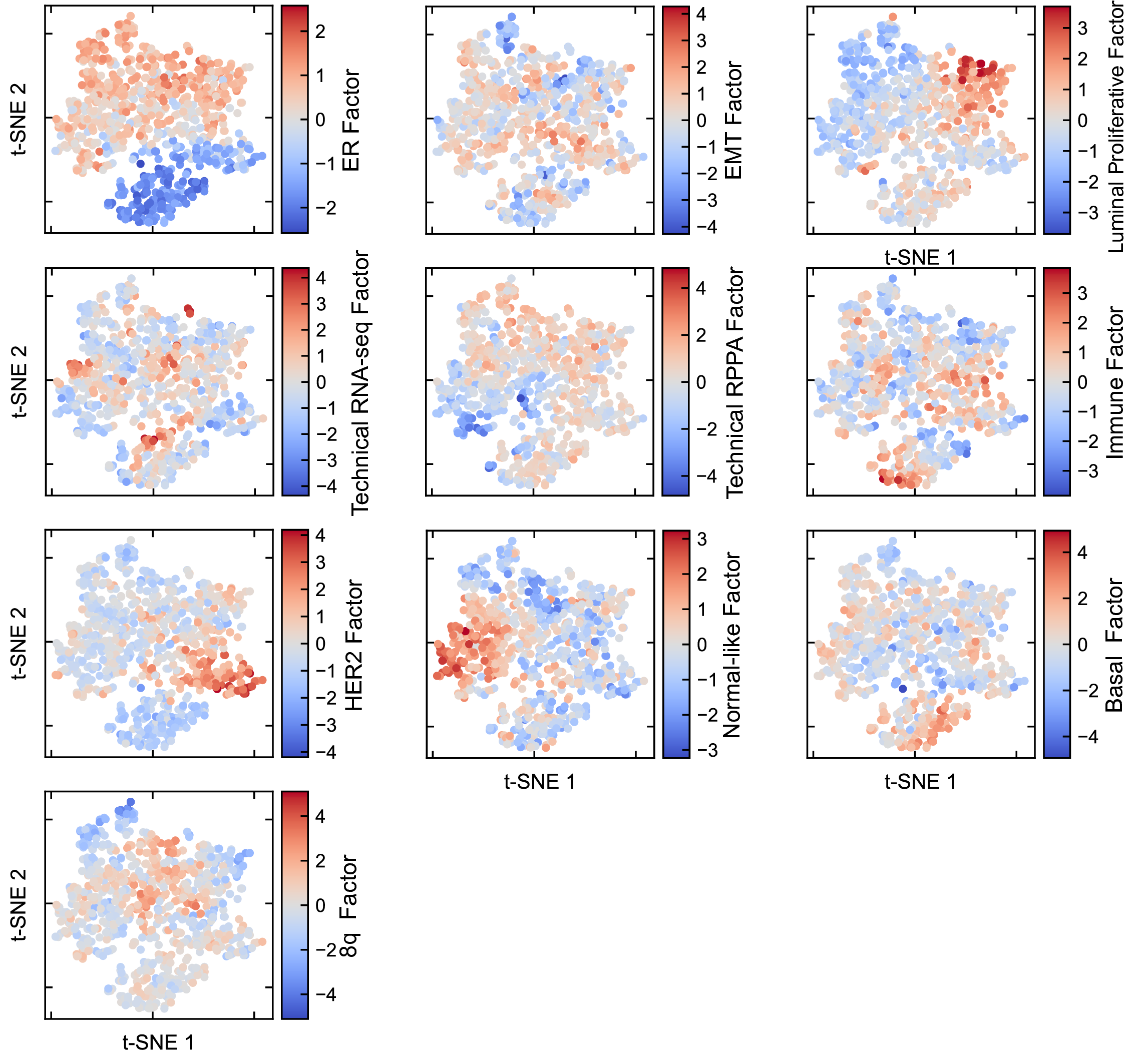

Supplement: S6 Fig — A selection of these is also shown in Fig 3B. (TIF) [file pcbi.1006520.s011.tif]

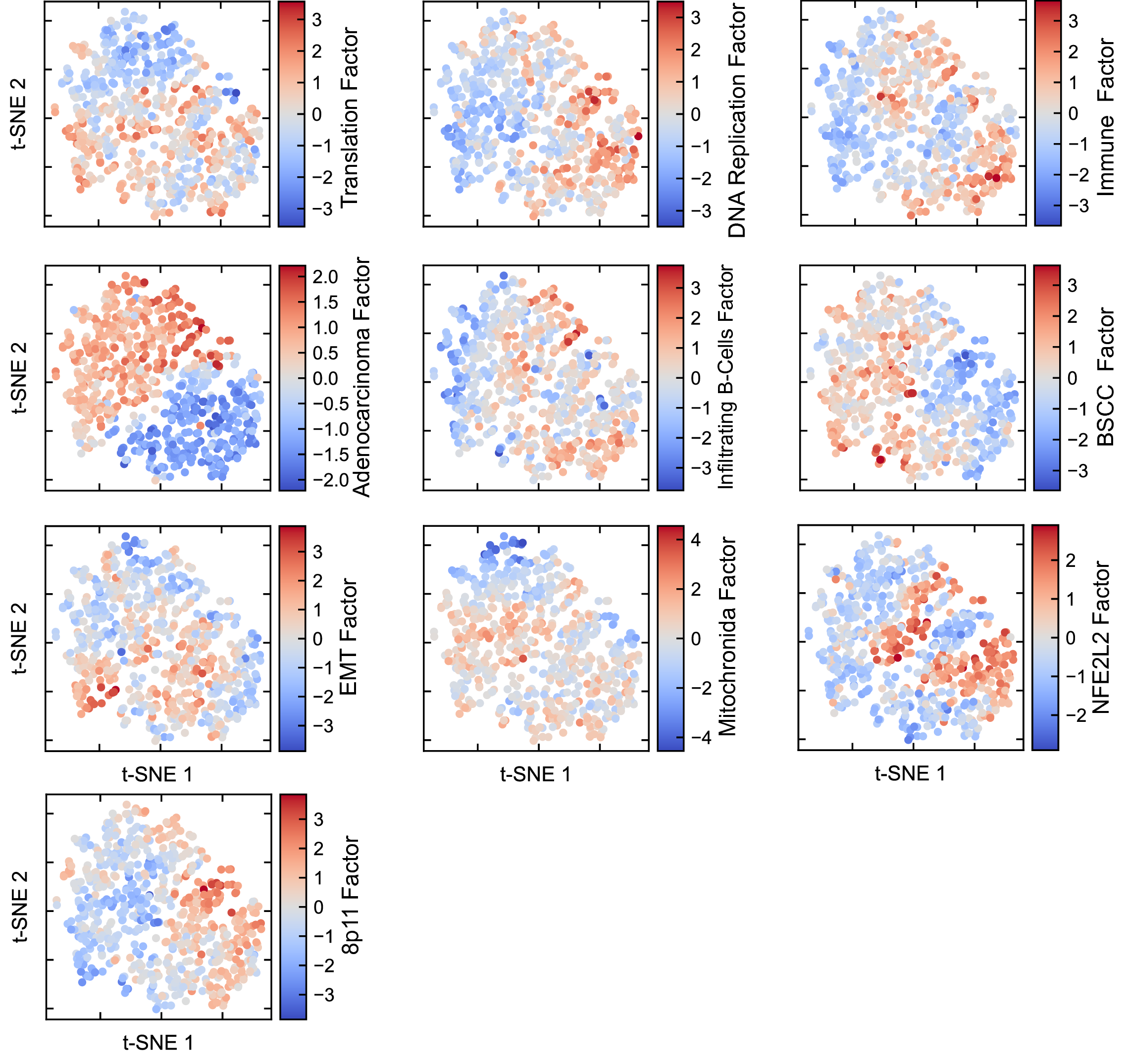

Supplement: S7 Fig — A selection of these is also shown in Fig 7B. (TIF) [file pcbi.1006520.s012.tif]

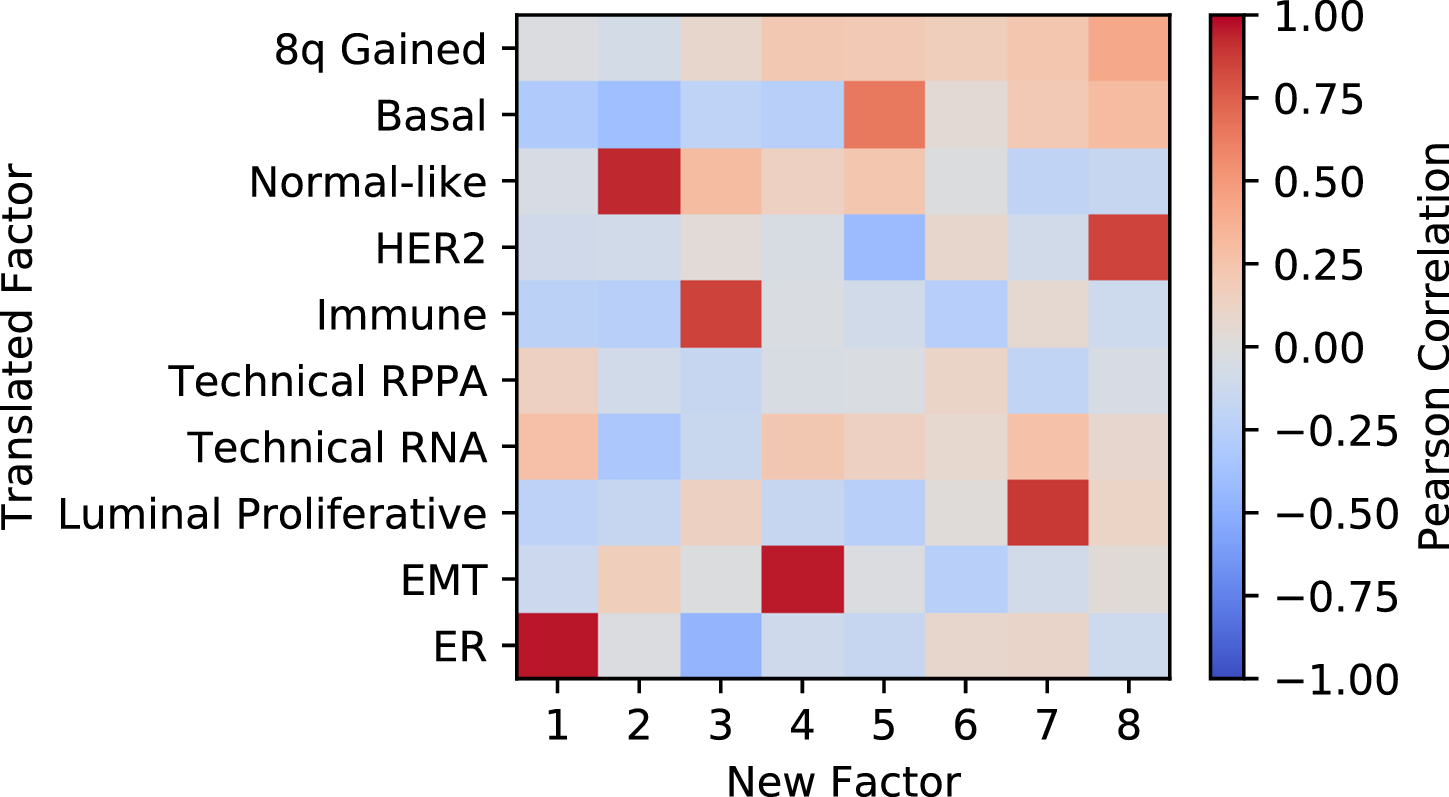

Supplement: S11 Fig — (TIF) [file pcbi.1006520.s016.tif]

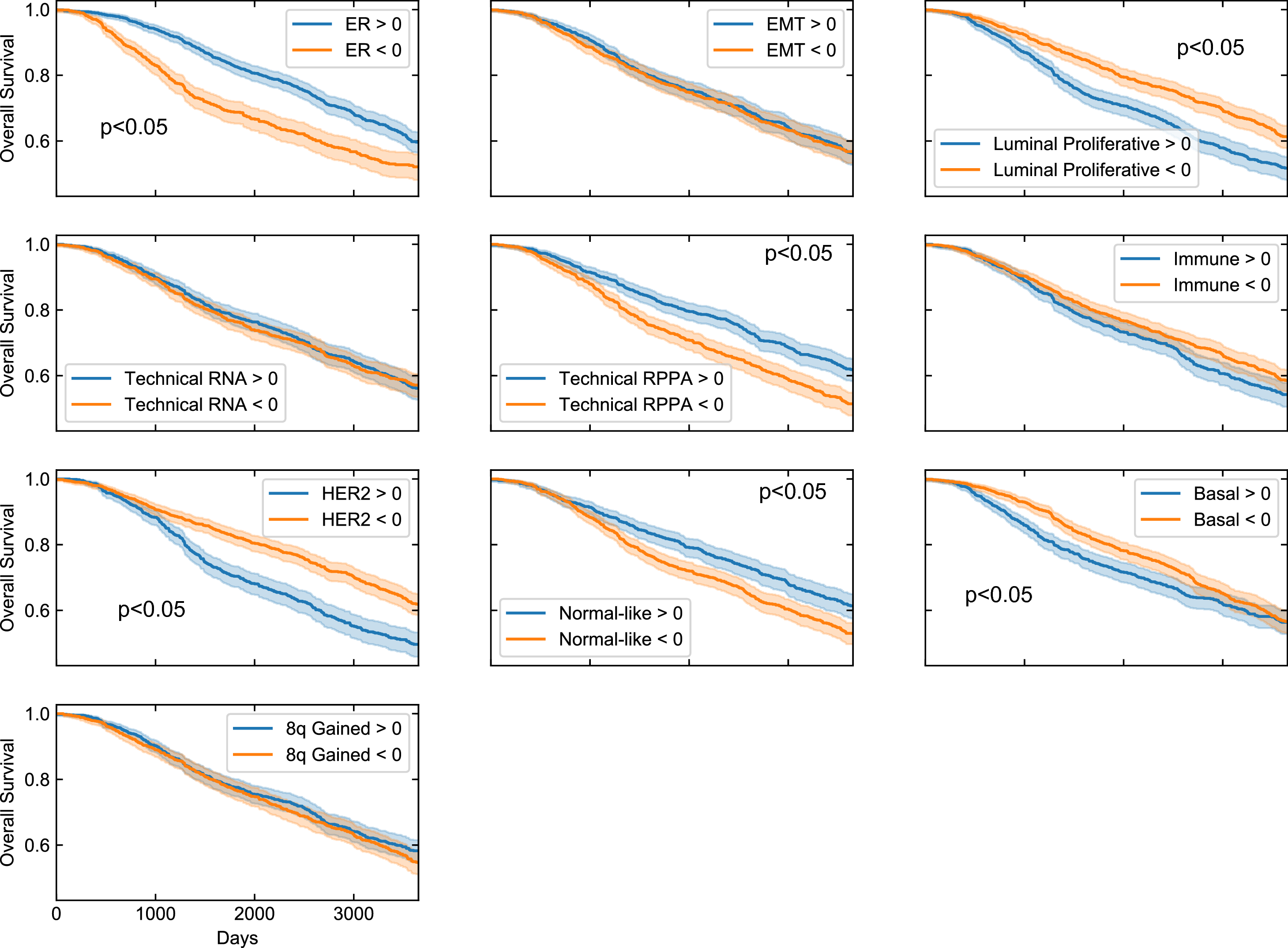

Supplement: S12 Fig — Signifance survival difference is assesed with the log-rank test. (TIF) [file pcbi.1006520.s017.tif]

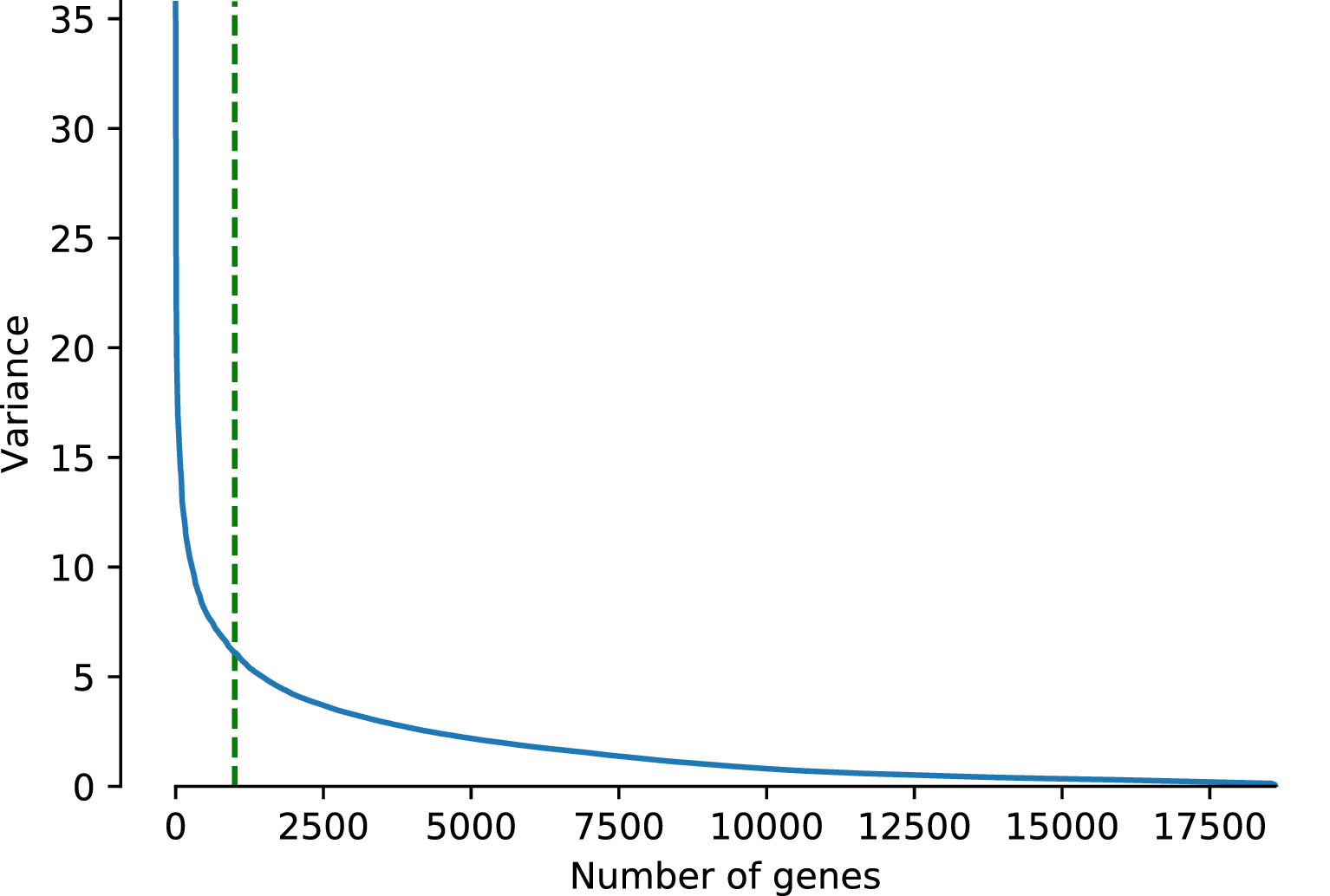

Supplement: S13 Fig — (TIF) [file pcbi.1006520.s018.tif]

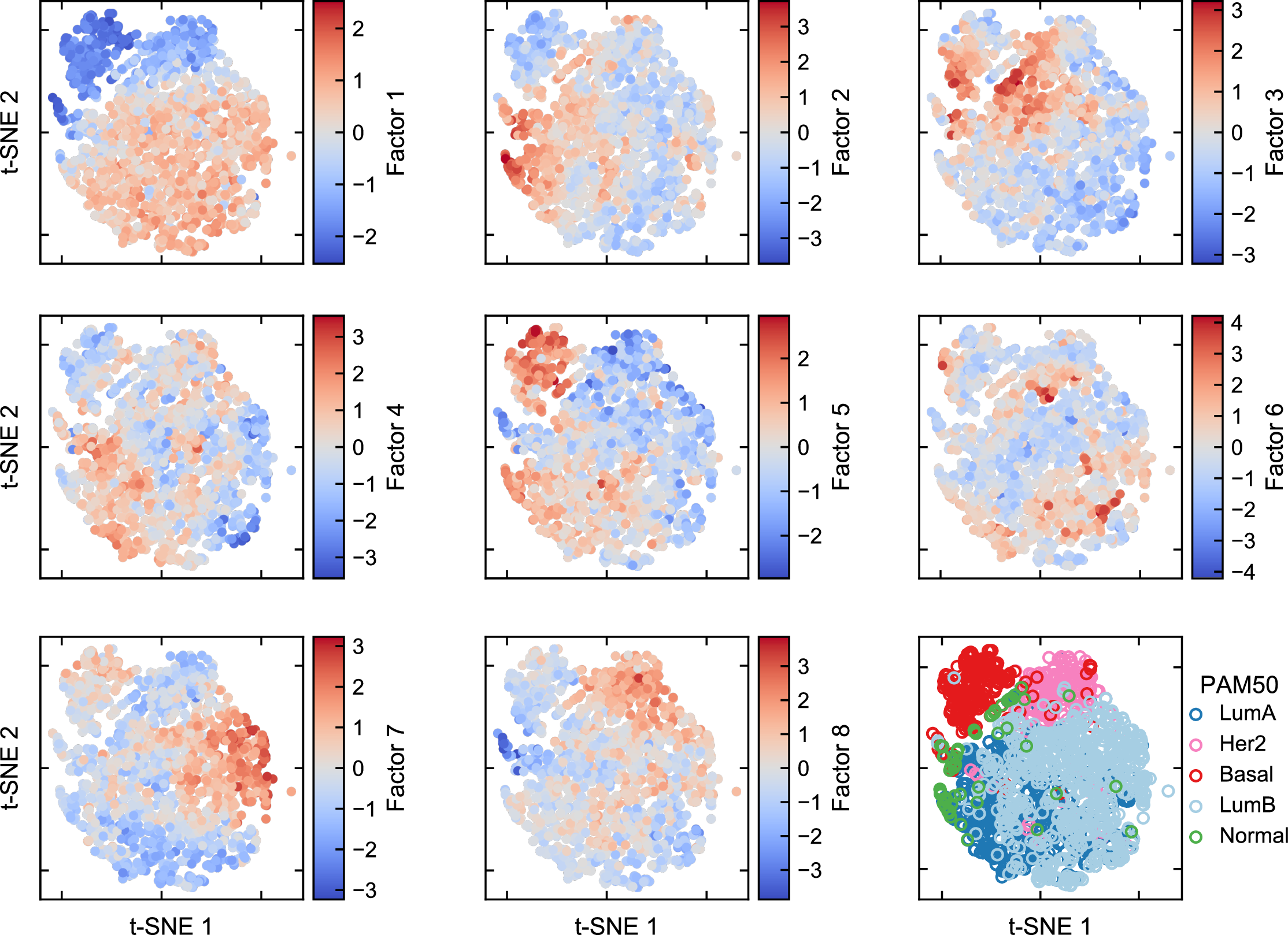

Supplement: S14 Fig — (TIF) [file pcbi.1006520.s019.tif]

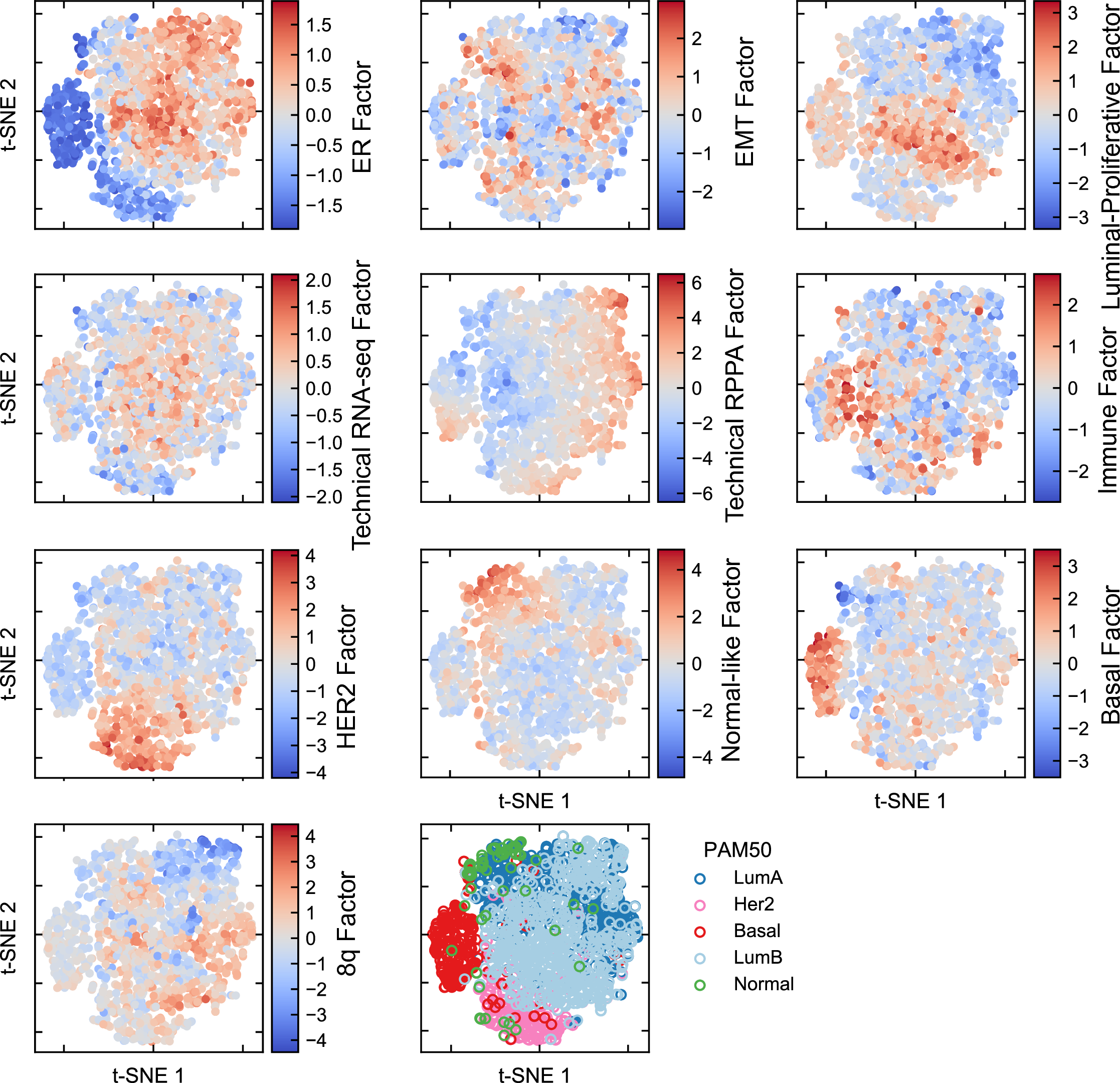

Supplement: S15 Fig — (TIF) [file pcbi.1006520.s020.tif]

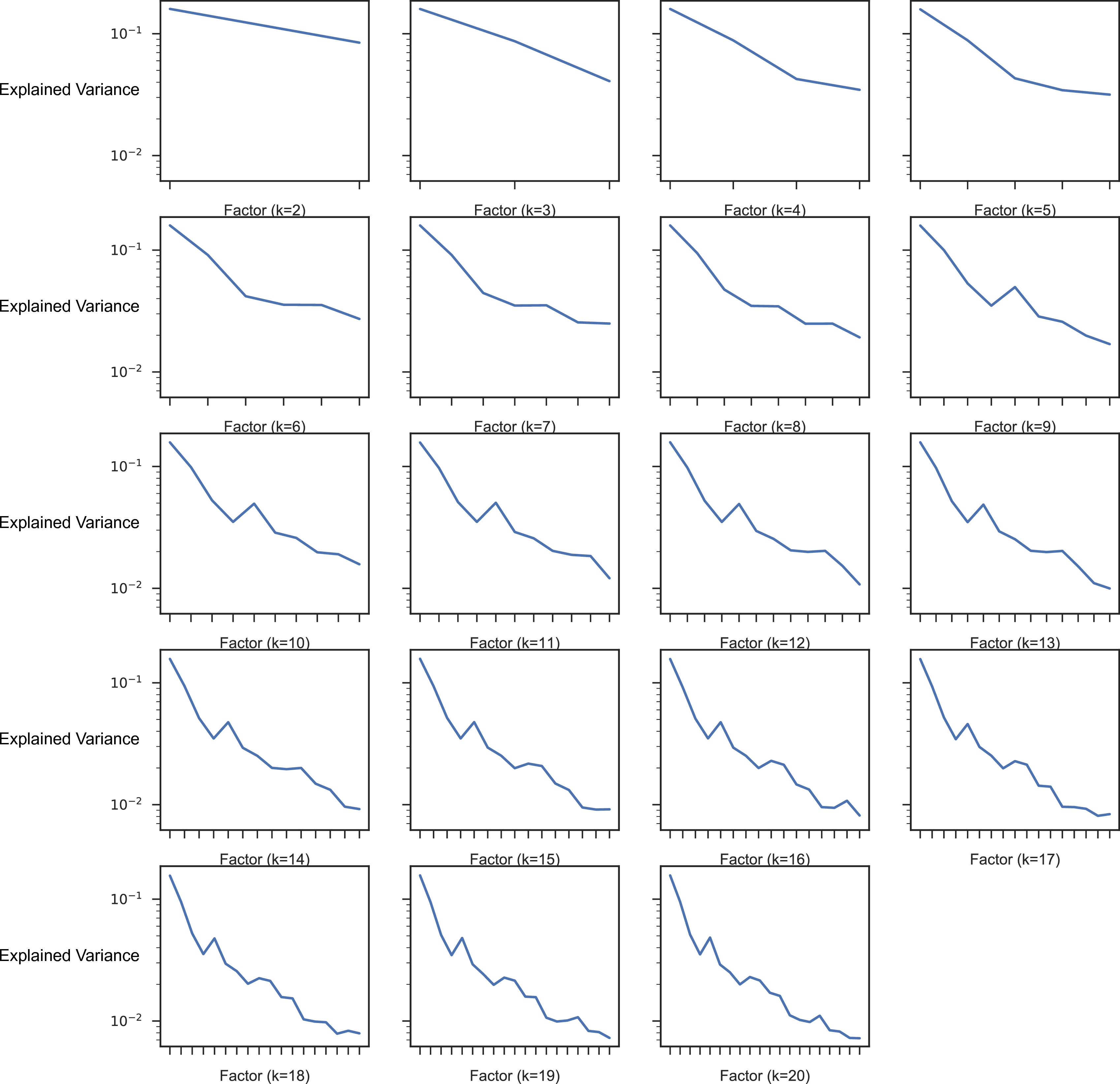

Supplement: S16 Fig — The models are the same as those shown in S2 Fig. (TIF) [file pcbi.1006520.s021.tif]
